# Long-term safety profile of opicapone in patients with Parkinson’s disease and early fluctuations

**DOI:** 10.3389/fneur.2026.1920359

**Published:** 2026-10-13

**Authors:** Joaquim J. Ferreira, Fabrizio Stocchi, Angelo Antonini, Olivier Rascol, Nicola Pavese, Georg Ebersbach, Jaime Kulisevsky, Günter Höglinger, Daniel Ramos, José Francisco Rocha, Helena C. Brigas, Joerg Holenz, Werner Poewe

**Affiliations:** 1 Laboratory of Clinical Pharmacology and Therapeutics, Faculdade de Medicina, Universidade Lisboa, Lisbon, Portugal; 2 CNS – Campus Neurológico, Torres Vedras, Portugal; 3 Institute for Research and Medical Care IRCCS San Raffaele, Roma, Italy; 4 Department of Neurology, San Raffaele University, Rome, Italy; 5 Neurodegenerative Disease Unit, Centre for Rare Neurological Diseases (ERN-RND), Department of Neuroscience, University of Padova, Padova, Italy; 6 IRCCS San Camillo Venice, Venice, Italy; 7 Department of Clinical Pharmacology and Neurosciences, Parkinson Expert Centre, Clinical Investigation Centre CIC1436, University Hospital of Toulouse, INSERM, University of Toulouse 3, Toulouse, France; 8 French Clinical Research Network for Parkinson's Disease and Movement Disorders (NS-PARK), Toulouse, France; 9 Clinical Ageing Research Unit, Newcastle University, Newcastle upon Tyne, United Kingdom; 10 Movement Disorders Hospital, Beelitz-Heilstätten, Germany; 11 Movement Disorders Unit, Neurology Department, Sant Pau Hospital, Barcelona, Spain; 12 Biomedical Research Institute (IIB-Sant Pau), Barcelona, Catalunya, Spain; 13 Center for Networked Biomedical Research in Neurodegenerative Diseases (CIBERNED), Madrid, Spain; 14 Department of Neurology, Ludwig-Maximilians-Universität Hospital, Ludwig-Maximilians-Universität, Munich, Germany; 15 German Center for Neurodegenerative Diseases, Munich, Germany; 16 Munich Cluster for Systems Neurology (SyNergy), Munich, Germany; 17 BIAL – Portela & Ca S.A., Coronado, Portugal; 18 Department of Neurology, Medical University of Innsbruck, Innsbruck, Austria

**Keywords:** clinical trial, long-term, motor fluctuations, opicapone, Parkinson’s disease, safety

## Abstract

**Background:**

Motor fluctuations frequently emerge within the first few years of levodopa treatment in Parkinson’s disease (PD), increasing symptom burden and treatment complexity. While opicapone is an established adjunct therapy for motor fluctuations, its safety profile when initiated early in the course of fluctuating disease remains incompletely characterized.

**Methods:**

This pooled *post-hoc* analysis integrated individual patient-level data from two randomized, double-blind, placebo-controlled trials of opicapone 50 mg and their open-label extensions. Analyses focused on patients with recent onset of motor fluctuations (≤2 years). Safety outcomes included the incidence, severity, timing, and evolution of treatment-emergent adverse events (TEAEs) during double-blind and up to 1 year of open-label exposure, with comparisons between earlier (OPC–OPC) and delayed (PLC–OPC) initiation.

**Results:**

A total of 269 patients with recent motor fluctuations were included, of whom 228 entered open-label treatment. During the double-blind phase, TEAEs were reported in 55.9% of patients receiving opicapone and 50.9% receiving placebo, with no statistically significant difference between groups. Adverse events were mainly mild to moderate and occurred predominantly within the first 2–3 weeks declining thereafter. Dyskinesia was the most frequent TEAE (10.2% vs. 3.6%, *p* = 0.09), occurring early and resolving in most patients, either spontaneously or following modest levodopa dose adjustment. Across the combined study period, overall TEAE incidence and cumulative dyskinesia rates were comparable between early and delayed initiation groups (15.3% vs. 15.5%, *p* = 1.0), with no increase over time.

**Conclusion:**

Opicapone demonstrated a favorable and consistent safety profile in patients with recent onset of motor fluctuations, with adverse events predominantly transient and manageable. Earlier initiation was not associated with an increased safety burden and showed numerically lower rates of overall TEAEs and dyskinesia compared with broader trial populations. These findings support the hypothesis that early optimization of levodopa therapy with opicapone does not accelerate the development of motor complications, supporting early COMT-inhibition in fluctuating PD.

## 1 Introduction

OFF symptoms represent a key milestone in the clinical course of Parkinson’s disease (PD), often emerging within the first few years of levodopa therapy and progressively increasing symptom burden and treatment complexity (1, 2). Around half of patients treated with levodopa develop motor fluctuations within approximately 5 years, with prevalence increasing substantially with longer follow-up (1, 3). Higher levodopa doses, younger age at onset, and female sex are consistently associated with an increased risk of motor complications (1, 4). Although catechol-O-methyltransferase (COMT) inhibitors have traditionally been reserved for patients with more advanced motor fluctuations, there is growing interest in approaches that optimize levodopa delivery earlier in the disease course, to improve symptom control while minimizing adverse events and the need for dose escalation (5–7).

Opicapone is a once-daily, peripherally selective COMT inhibitor that prolongs levodopa bioavailability and optimizes its delivery, thereby reducing end-of-dose deterioration (5). Its efficacy and safety have been well established in patients with fluctuations (8, 9), where adverse events are predominantly dopaminergic, occur early after initiation, and are generally manageable with levodopa dose adjustment (10). However, these data largely derive from populations with more advanced disease; evidence in earlier stages remains limited, although emerging observational data suggest potential for a more favorable tolerability profile compared with use in more advanced stages (7, 11–13).

We recently reported the long-term efficacy of opicapone in patients with early motor fluctuations (≤2 years’ duration), based on a subgroup analysis of the pooled BIPARK trials, demonstrating clinically meaningful improvements with minimal levodopa dose escalation (14). Here, we present the accompanying safety and tolerability analysis in this same population. The main objectives of the present *post-hoc* analysis were to evaluate the safety of opicapone 50 mg versus placebo in patients with early onset of motor fluctuations (≤2 years) and to assess long-term safety over 1 year in this population. By comparing safety outcomes in patients treated with opicapone throughout the double-blind and open-label phases with those who switched from placebo to opicapone, we also explored whether earlier initiation influences the emergence and pattern of dopaminergic adverse events, particularly dyskinesia. In addition, given the potential for mild psychiatric, cognitive, and sleep-related comorbidities to influence PD treatment tolerability and persistence, we sought to explore safety outcomes in patients with early motor fluctuations and these coexisting mild conditions, recognizing that patients with more severe comorbidities were not represented in the studies.

## 2 Materials and methods

BIPARK-I (8) and BIPARK-II (9) were randomized, double-blind, placebo-controlled trials of 14–15 weeks’ duration, with methodological details reported previously. Both studies evaluated once-daily opicapone 50 mg, administered in the evening at least 1 h after the final daily levodopa/DDCI dose, versus placebo. Lower doses (5 mg in BIPARK-I and 25 mg in both studies) and the entacapone arm in BIPARK-I were not considered in the present analysis. Both double-blind trials included subsequent open-label extension phases with follow-up of up to 1 year (9, 15).

The double-blind and open-label extension studies were approved by institutional review boards at all participating centers and were conducted in accordance with the Declaration of Helsinki and International Conference on Harmonization Good Clinical Practice guidelines. All patients provided written informed consent prior to initiation of study procedures.

### 2.1 Participants and study designs

In brief, both trials enrolled adults aged 30–83 years with idiopathic PD of at least 3 years’ duration and a modified Hoehn and Yahr stage of 1–3 during ON periods (8, 9). Participants were required to be receiving levodopa administered in 3–8 daily doses for a minimum of 1 year and to experience end-of-dose motor fluctuations with ≥1.5 h of daily OFF time, excluding morning akinesia, to reduce variability and allow reliable assessment of daytime OFF periods. Key exclusion criteria across both studies included a Unified Parkinson’s Disease Rating Scale (UPDRS) item 33 dyskinesia disability score >3, severe or unpredictable OFF periods, and any prior surgical intervention or deep brain stimulation for PD. Patients with clinically significant or unstable cardiovascular or psychiatric conditions (including major depression, dementia, impulse control disorders, or suicidal ideation), clinically significant liver disease, or transaminase levels exceeding twice the upper limit of normal were excluded.

In the double-blind phase, reductions in the daily dose, but not frequency, of Levodopa/DDCI could be made within the first 4 weeks of opicapone initiation but were not permitted thereafter. Concomitant PD treatments were permitted if stable, with the exception of tolcapone, apomorphine (withdrawn at least 1 month prior to baseline), and entacapone (unless provided as part of the BIPARK-I protocol).

Immediately following completion of the double-blind period, patients entered open-label extension phases lasting up to 52 weeks. All participants initiated open-label treatment with opicapone 25 mg, with the option to up-titrate to 50 mg to improve symptom control. In the event of dopaminergic adverse events, levodopa dose adjustments were prioritized, followed by down-titration of opicapone if required.

### 2.2 Outcome analysis

This pooled post-hoc analysis integrated individual participant-level data and included participants from either study who were randomized to placebo or opicapone 50 mg, received ≥1 dose of study medication, had ≥1 post-baseline OFF-time assessment, continued opicapone treatment into the open-label extension phases, and had investigator-judged motor fluctuation onset within the 2 years preceding the double-blind screening visit. The primary outcome was the safety and tolerability of opicapone 50 mg compared with placebo during the double-blind phase, assessed through the incidence, severity, and timing of treatment-emergent adverse events (TEAEs). Other outcomes included the long-term safety profile during and up to 1 year of open-label exposure, and comparisons of safety outcomes between patients with earlier versus delayed initiation of opicapone across the combined double-blind and open-label periods. Standard adverse event reporting included overall TEAEs, severe TEAEs, serious TEAEs, TEAEs leading to treatment discontinuation, and TEAEs considered potentially related to treatment. Elevations in blood creatine phosphokinase (CPK) were evaluated based on laboratory analysis with mild elevations defined as 1–3 times the upper normal limit (UNL), moderate elevations as 3–10 times the UNL, and severe elevations as >10 times UNL.

For key adverse events, the time course of onset, cumulative incidence, severity, and outcome (recovery or persistence) were evaluated, including during the transition from double-blind to open-label treatment. To explore the impact of earlier versus delayed exposure to opicapone, adverse events were analyzed across the full study duration, comparing patients who received opicapone from the start of the double-blind phase (designated OPC–OPC) with those who switched from double-blind placebo to open-label treatment with opicapone (designated PLC–OPC).

Differences between the two groups were assessed using Pearson’s chi-square test with Yates’ continuity correction or Fisher’s exact test, as appropriate (16). Fisher’s exact test was employed when the assumptions of the chi-square test were violated, namely when expected cell frequencies were low. The assumptions for Pearson’s chi-square test require that all expected cell counts exceed 1 and that no more than 20% of cells have expected frequency below 5. Given the small number of observed events and the absence of adjustment for multiple comparisons, statistical significance should be interpreted cautiously. The analyses were exploratory and hypothesis-generating rather than confirmatory. For findings with nominal statistical significance (*p* < 0.05), *post-hoc* power analyses were conducted to evaluate the likelihood of detecting the observed effects.

Although BIPARK I and II excluded patients with major neuropsychiatric and sleep comorbidities, a separate exploratory subgroup analysis was conducted in patients with central nervous system (CNS) comorbidities (psychiatric and nervous system disorders, according to the System Organ Classes of the Medical Dictionary for Regulatory Activities). Within this, psychosis, cognitive decline, anxiety, depressive events, and sleep disorders among others were considered as events of special interest to COMT inhibitors or PD (10). Safety outcomes in this subgroup were summarized separately for the double-blind phase and for the open-label exposure, with a focus on treatment-related adverse events.

All safety analyses were descriptive, based on the observed-case dataset. Analyses were performed using R version 4.5.1.

## 3 Results

### 3.1 Study population

Of the 535 participants randomized to receive placebo or opicapone 50 mg in BIPARK-I and BIPARK-II, 269 had investigator-judged onset of motor fluctuations within the 2 years prior to study entry. Among these, 228 entered the open-label phase and comprised the safety population (Supplementary Figure 1). Baseline demographic and clinical characteristics were well balanced between treatment groups at the start of the double-blind phase (Table 1). Despite the relatively recent onset of motor fluctuations (mean duration 1.0 year), patients experienced a substantial motor symptom burden, with a mean of 6.3 h of OFF-time per day at baseline. Most participants were receiving levodopa in 3–5 daily doses, with a mean levodopa exposure of ~4.6–4.9 years and a mean daily levodopa dose of ~635–649 mg/day at double-blind baseline (corresponding to a mean LEDD of ~751–761 mg/day). Most patients were treated with at least one concomitant antiparkinsonian medication, most commonly dopamine agonists, with additional use of MAO-B inhibitors, amantadine and, less frequently, anticholinergics. Ongoing comorbidities were frequent, most commonly vascular disorders, reported in approximately half of patients (49–53%), with nervous system disorders also present in around one-quarter to one-third of participants (26–32%) across treatment groups (Table 1).

**Table 1:** Participant demographics and clinical characteristics at double-blind baseline.

Characteristic	Opicapone 50 mg (*n* = 118)	Placebo (*n* = 110)
Demographics		
Male	78 (66.1%)	62 (56.4%)
Age, years	64.7 ± 9.0	63.9 ± 8.8
Clinical history		
Time since PD diagnosis, years	6.0 ± 2.9	6.1 ± 2.7
Modified Hoehn & Yahr at ON, stage	2.4 ± 0.5	2.4 ± 0.5
UPDRS Part III (motor) score	25.7 ± 11.9	26.3 ± 12.0
Motor complication profile		
Time since motor fluctuation onset, years	1.0 ± 0.6	1.0 ± 0.6
Daily OFF-time, hours	6.3 ± 2.1	6.3 ± 2.1
Daily Good ON-time, hours	9.5 ± 2.2	9.6 ± 2.0
ON-time with troublesome dyskinesia, hours	0.3 ± 0.8	0.4 ± 1.0
Presence of dyskinesia	41 (34.7%)	40 (36.4%)
Levodopa use		
Levodopa duration, years	4.6 ± 3.0	4.9 ± 2.6
Levodopa dose at double-blind baseline, mg/day	635 ± 319	649 ± 305
LEDD^$^ at double-blind baseline, mg/day	751 ± 331	761 ± 332
LEDD^$^ at open-label baseline, mg/day	1,059 ± 471^£^	750 ± 312
Concomitant PD medication use		
Levodopa monotherapy*	23 (19.5%)	22 (20.0%)
Dopamine agonists	82 (69.5%)	73 (66.4%)
MAO-B inhibitor	23 (19.5%)	19 (17.3%)
Amantadine	27 (22.9%)	20 (18.2%)
Anticholinergics	5 (4.2%)	11 (10.0%)
Ongoing medical history		
Any comorbidity	111 (94.1%)	98 (89.1%)
Ongoing comorbidity in >20% of patients per arm		
Vascular disorders	58 (49.2%)	58 (52.7%)
Musculoskeletal and connective tissue disorders	51 (43.2%)	48 (43.6%)
Metabolism and nutrition disorders	46 (39.0%)	37 (33.6%)
Nervous system disorders	31 (26.3%)	35 (31.8%)
Psychiatric disorders	29 (24.6%)	23 (20.9%)
Cardiac disorders	27 (22.9%)	24 (21.8%)
Gastrointestinal disorders	24 (20.3%)	31 (28.2%)

Data are mean ± SD or n (%). ^$^LEDD calculated considering MAO-B inhibitors and dopamine agonists. ^£^Considering OPC for LEDD calculation. *Defined as without dopamine agonists, MAO-B inhibitors, amantadine or anticholinergics.

### 3.2 Double-blind phase

During the double-blind phase, TEAEs were reported in 55.9% of patients receiving opicapone 50 mg and 50.9% of those receiving placebo, with no significant difference between groups (*p* = 0.53; Table 2). Most TEAEs were mild to moderate in severity. Severe TEAEs were uncommon and occurred at similar rates in both treatment groups (3.4% with opicapone vs. 3.6% with placebo), as were serious TEAEs (2.5% vs. 1.8%, respectively). TEAEs considered potentially related to study treatment occurred in 30.5% of patients receiving opicapone 50 mg and 20.0% of those receiving placebo, although this difference was not statistically significant (*p* = 0.10); no serious treatment-related TEAEs were reported in the opicapone group. Blood CPK increased was reported more frequently with opicapone than placebo among all TEAEs (6 [5.1%] vs. 0; *p* = 0.03; *post-hoc* power estimate, 55.9%). However, no significant between-group differences were observed for treatment-related blood CPK elevations (3 [2.5%] vs. 0 [0.0%]; *p* = 0.25), during the open-label phase (7 [5.9%] vs. 6 [5.5%]; *p* = 1.00), or across the combined exposure period (13 [11.0%] vs. 6 [5.5%]; *p* = 0.20).

**Table 2:** Safety and tolerability in the double-blind phase.

Adverse event category	Opicapone 50 mg *n* = 118	Placebo *n* = 110	*p*-value
All TEAEs	66 (55.9%)	56 (50.9%)	0.53^±^
Mild	58 (49.2%)	45 (40.9%)	0.26^±^
Moderate	30 (25.4%)	16 (14.5%)	0.06^±^
Severe	4 (3.4%)	4 (3.6%)	1.00*^$^*
Serious	3 (2.5%)	2 (1.8%)	1.00*^$^*
Leading to discontinuation	0	0	-
All Related TEAEs	36 (30.5%)	22 (20.0%)	0.10^±^
Serious	0	2 (1.8%)^#^	0.23*^$^*
TEAEs occurring in ≥5% in any arm			
Dyskinesia	12 (10.2%)	4 (3.6%)	0.09*^$^*
Constipation	6 (5.1%)	2 (1.8%)	0.28*^$^*
Nausea	3 (2.5%)	5 (4.5%)	0.49*^$^*
Blood CPK increased	6 (5.1%)	0	0.03*^$∆^*(*post-hoc* power 55.9%*^∆^*)
Related TEAEs occurring in ≥3% in any arm, n (%)			
Dyskinesia	11 (9.3%)	4 (3.6%)	0.14^±^
Dry mouth	3 (2.5%)	2 (1.8%)	1.00*^$^*
Nausea	2 (1.7%)	3 (2.7%)	0.67*^$^*
Insomnia	4 (3.4%)	0	0.12*^$^*
Blood CPK increased	3 (2.5%)	0	0.25*^$^*

#Radius fracture, *n* = 1; Superficial spreading melanoma stage unspecified, *n* = 1; Statistical comparisons were exploratory and not adjusted for multiplicity; given the number of tests performed and the limited sample size, *p*-values should not be interpreted as confirmatory evidence of a treatment difference [*p*-values were calculated using ^±^Chi-square test with Yates’ continuity correction or ^$^Fisher’s exact test. ^∆^For statistically significant findings (*p* < 0.05), *post-hoc* power analysis was performed to assess the probability of detecting the observed effect].

Overall TEAEs, including those considered potentially treatment-related, were predominantly transient and occurred early during the double-blind phase (Figures 1a,b). Onset-by-week and cumulative incidence analyses indicated that the majority of events occurred within the first 2 to 3 weeks of treatment and diminished substantially thereafter. Fifteen patients (mean daily levodopa dose 850 mg; mean daily LEDD 970 mg) experienced dyskinesia events during the double-blind phase. Dyskinesia occurred predominantly within the first 2 weeks of treatment after which events were infrequent and cumulative incidence stabilized (Figure 1c). Dyskinesia resolved in most patients (12/15), with six reducing their levodopa dose (mean reduction 18%) and six recovering without levodopa dose adjustment. The majority of dyskinesia events were reported as mild-to-moderate in severity and occurred within the first 2 weeks of treatment. In both cases where dyskinesia persisted, dyskinesia was already present at baseline; outcome data were missing for one patient. Detailed characterization of dyskinesia phenomenology (e.g., focal versus generalized dyskinesia) was not systematically collected as part of TEAE reporting.

**Figure 1:**
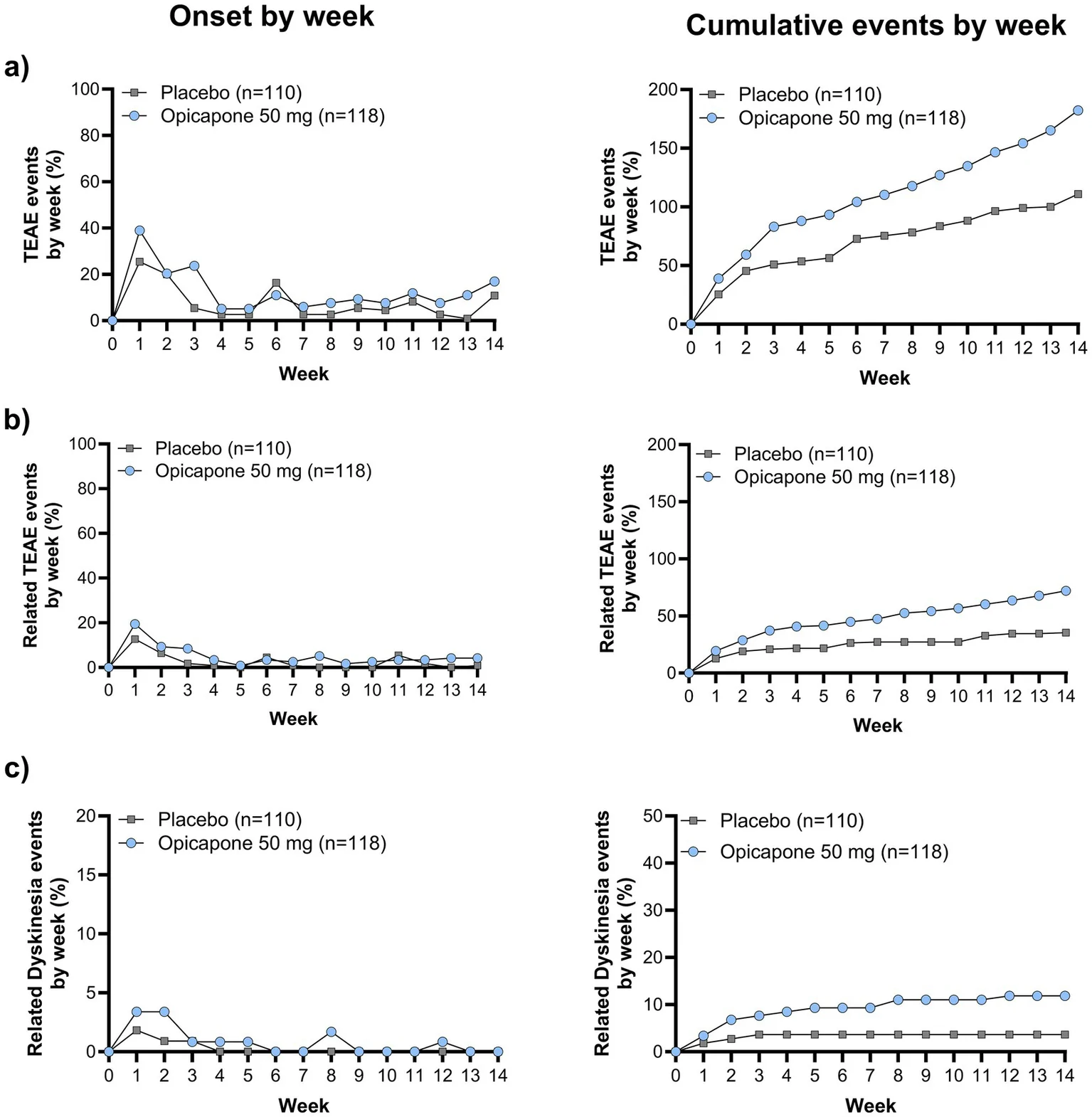
Time course of adverse events during the double-blind phase (event onset) and cumulative incidence for **(a)** Overall treatment-emergent adverse events, **(b)** Treatment-related TEAEs, and **(c)** Dyskinesia in patients with onset of motor fluctuations ≤2 years. Graphs display the total number of events, and a single patient may have reported more than one event. Potentially related defined as definite, possible, probable, or with missing information.

### 3.3 Open-label phase

During the open-label treatment phase, TEAEs were reported in 71.2% of patients who received opicapone throughout the study (OPC–OPC) and 70.0% of those who switched from placebo to opicapone (PLC–OPC), with no significant difference between groups (*p* = 0.96; Table 3). Most TEAEs were mild or moderate in severity, and serious TEAEs remained infrequent. The most commonly reported TEAEs during open-label treatment were dyskinesia (9.3% OPC–OPC vs. 15.5% PLC–OPC, *p* = 0.23), worsening of Parkinsonism (10.2% vs. 8.2%, *p* = 0.77), and drug effect decreased (6.8% vs. 7.3%, *p* = 1.0).

**Table 3:** Safety and tolerability across open-label period and combined double-blind and open-label phases.

Adverse event category	Open-label extension			Combined double-blind phase and open-label extension			
	OPC–OPC *n* = 118	PLC–OPC *n* = 110	*p*-value	OPC–OPC *n* = 118	PLC–OPC *n* = 110	All*N* = 228	*p*-value
All TEAEs	85 (71.2%)	77 (70.0%)	0.96^±^	93 (78.8%)	88 (80.0%)	181 (79.4%)	0.95^±^
Mild	67 (56.8%)	68 (61.8%)	0.52^±^	82 (69.5%)	81 (73.6%)	163 (71.5%)	0.59^±^
Moderate	49 (41.5%)	42 (38.2%)	0.70^±^	56 (47.5%)	47 (42.7%)	103 (45.2%)	0.56^±^
Serious	11 (9.3%)	9 (8.2%)	0.94^±^	14 (11.9%)	11 (10.0%)	25 (11.0%)	0.81^±^
Leading to discontinuation	6 (5.1%)	0	0.03*^$^* (55.9%*^∆^*)	6 (5.1%)	0	6 (2.6%)	0.03*^$^* (55.9%*^∆^*)
Related TEAEs	46 (39.0%)	39 (35.5%)	0.68^±^	59 (50.0%)	46 (41.8%)	105 (46.1%)	0.27^**±**^
Serious	3 (2.5%)^&^	0	0.25*^$^*	3 (2.5%)^&^	2 (1.8%)^#^	5 (2.2%)	1.00*^$^*
Leading to discontinuation	4 (3.4%)*	0	0.12*^$^*	4 (3.4%)*	0	4 (1.8%)	0.12*^$^*
Deaths	0	0	-	0	0	0	-
TEAEs occurring in ≥5% in any arm							
Dyskinesia	11 (9.3%)	17 (15.5%)	0.23^±^	23 (19.5%)	21 (19.1%)	44 (19.3%)	1.00^±^
Worsening of parkinsonism	12 (10.2%)	9 (8.2%)	0.77^±^	14 (11.9%)	10 (9.1%)	24 (10.5%)	0.20^±^
Drug effect decreased	8 (6.8%)	8 (7.3%)	1.00^±^	10 (8.5%)	9 (8.2%)	19 (8.3%)	0.56^±^
Blood CPK increased	7 (5.9%)	6 (5.5%)	1.00^±^	13 (11%)	6 (5.5%)	19 (8.3%)	0.20^±^
Insomnia	7 (5.9%)	7 (6.4%)	1.00^±^	11 (9.3%)	7 (6.4%)	18 (7.9%)	0.56^±^
Nausea	3 (2.5%)	6 (5.5%)	0.32*^$^*	6 (5.1%)	11 (10.0%)	17 (7.5%)	0.25^±^
Constipation	4 (3.4%)	3 (2.7%)	1.00*^$^*	10 (8.5%)	5 (4.5%)	15 (6.6%)	0.35^±^
Fall	6 (5.1%)	6 (5.5%)	1.00^±^	6 (5.1%)	9 (8.2%)	15 (6.6%)	0.50^±^
Tremor	7 (5.9%)	4 (3.6%)	0.62^±^	7 (5.9%)	6 (5.5%)	13 (5.7%)	1.00^±^
Hypertension	5 (4.2%)	6 (5.5%)	0.91^±^	9 (7.6%)	7 (6.4%)	16 (7.0%)	0.91^±^
Back pain	2 (1.7%)	8 (7.3%)	0.05*^$^*	3 (2.5%)	11 (10.0%)	14 (6.1%)	0.04^±^ (67.6%)*^∆^*
Weight decrease	5 (4.2%)	4 (3.6%)	1.00*^$^*	9 (7.6%)	4 (3.6%)	13 (5.7%)	0.31^±^
Headache	4 (3.4%)	2 (1.8%)	0.68*^$^*	7 (5.9%)	6 (5.5%)	13 (5.7%)	1.00^±^
Depression	3 (2.5%)	6 (5.5%)	0.32*^$^*	5 (4.2%)	7 (6.4%)	12 (5.3%)	0.67^±^
Related TEAEs occurring in ≥3%							
Dyskinesia	10 (8.5%)	15 (13.6%)	0.30^±^	18 (15.3%)	17 (15.5%)	35 (15.4%)	1.00^±^
Blood CPK increased	5 (4.2%)	4 (3.6%)	1.00*^$^*	8 (6.8%)	4 (3.6%)	12 (5.3%)	0.44^±^
Insomnia	2 (1.7%)	4 (3.6%)	0.43*^$^*	6 (5.1%)	4 (3.6%)	10 (4.4%)	0.75*^$^*
Nausea	2 (1.7%)	4 (3.6%)	0.43*^$^*	3 (2.5%)	6 (5.5%)	9 (3.9%)	0.32*^$^*
Hallucinations (including visual)	5 (4.2%)	2 (1.8%)	0.45*^$^*	7 (5.9%)	2 (1.8%)	9 (3.9%)	0.17*^$^*
Weight decrease	3 (2.5%)	3 (2.7%)	1.00*^$^*	5 (4.2%)	3 (2.7%)	8 (3.5%)	0.72*^$^*
Dry mouth	1 (0.8%)	3 (2.7%)	0.35*^$^*	4 (3.4%)	4 (3.6%)	8 (3.5%)	1.00*^$^*
Worsening of parkinsonism	2 (1.7%)	5 (4.5%)	0.27*^$^*	2 (1.7%)	5 (4.5%)	7 (3.1%)	0.27*^$^*
Drug effect decreased	4 (3.4%)	2 (1.8%)	0.68*^$^*	4 (3.4%)	2 (1.8%)	6 (2.6%)	0.68*^$^*

^&^atrioventricular block complete, epilepsy, head injury, wrist fracture; ^#^Radius Fracture, *n* = 1; Superficial spreading melanoma stage unspecified, *n* = 1; *atrioventricular block complete, head injury, Parkinson’s disease, hallucination. Statistical comparisons were exploratory and not adjusted for multiplicity; given the number of tests performed and the limited sample size, *p*-values should not be interpreted as confirmatory evidence of a treatment difference [*p*-value were calculated using ^±^Chi-square test with Yates’ continuity correction or ^$^Fisher’s exact test. ^∆^For statistically significant findings (*p* < 0.05), *post-hoc* power analysis was performed to assess the probability of detecting the observed effect].

TEAEs considered potentially related to treatment occurred in 39.0% of OPC–OPC patients and 35.5% of PLC–OPC patients, with no significant difference between groups (*p* = 0.68). Potentially related serious TEAEs (2.5%) and treatment-related discontinuations in 4 patients (3.4%) were reported in the OPC–OPC arm (atrioventricular block complete, head injury, Parkinson’s disease, hallucination) and no patients in the PLC-OPC arm, although the between-group differences were not statistically significant (*p* = 0.25 and *p* = 0.12, respectively). Dyskinesia was again the most frequent treatment-related TEAE (8.5% OPC–OPC vs. 13.6% PLC–OPC), with a numerically higher incidence among patients who switched from placebo, although this difference was not significant (*p* = 0.30). As in the double-blind phase, patients with dyskinesia (versus those without) reported as a TEAE had more advanced disease, higher prevalence of dyskinesia at baseline, and higher therapeutic burden, with a longer PD duration, higher LEDD - driven by higher levodopa doses and a greater use of dopamine agonists and MAO-B inhibitors. No deaths were reported in either group. Consistent with the double-blind findings, TEAEs, including treatment-related events and dyskinesia, were generally transient over open-label follow-up (Figure 2).

**Figure 2:**
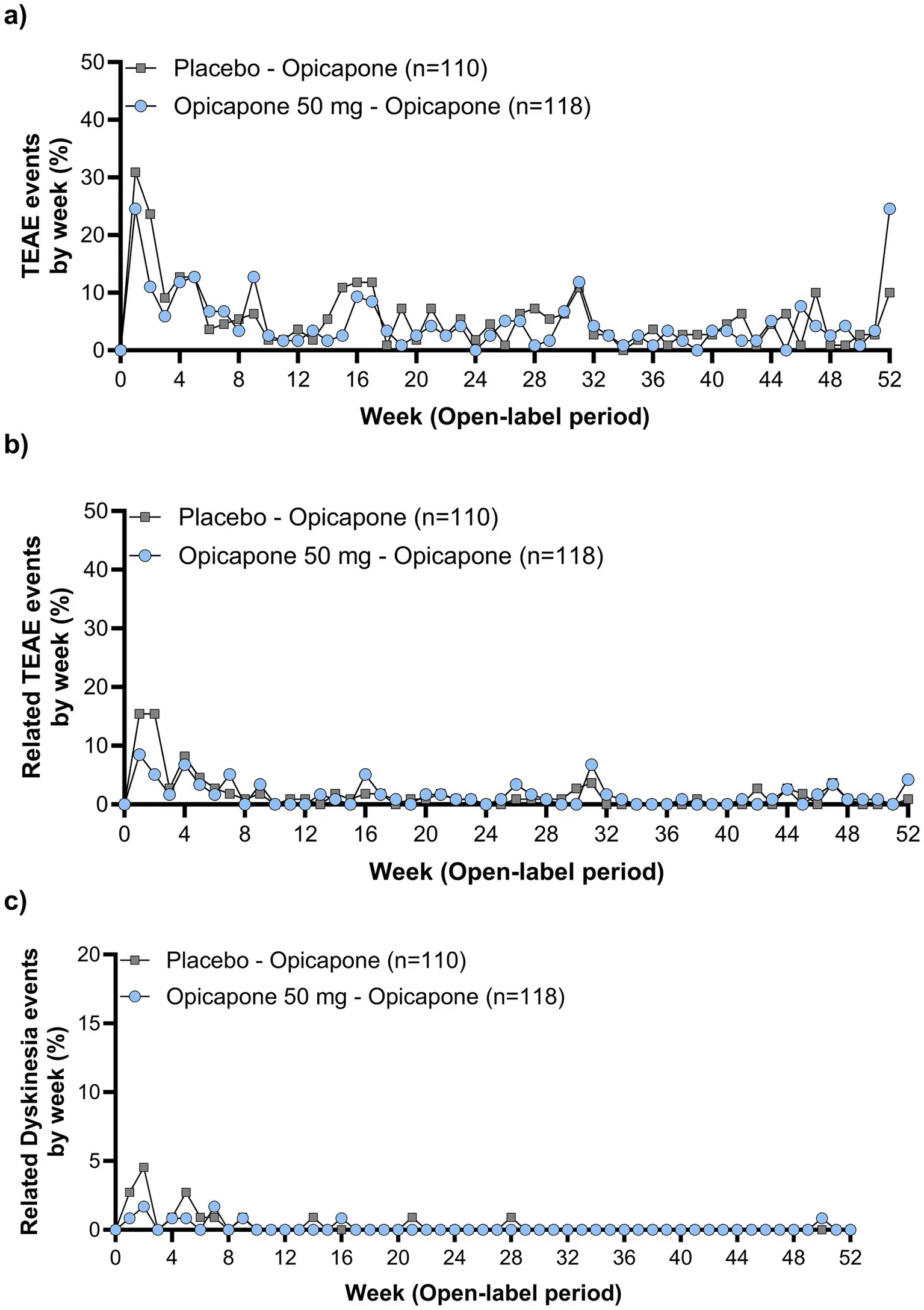
Time course of adverse events during the open-label phase (event onset) for **(a)** overall treatment-emergent adverse events, **(b)** treatment-related TEAEs, and **(c)** treatment-related dyskinesia in patients with onset of motor fluctuations ≤2 years. Graphs display the total number of events, and a single patient may have reported more than one event. Potentially related defined as definite, possible, probable or with missing information.

### 3.4 The safety and tolerability of early vs. later initiation of opicapone

To assess whether earlier versus delayed initiation of opicapone influenced long-term safety and tolerability, TEAEs were evaluated across the combined double-blind and open-label periods. The overall incidence of treatment-emergent adverse events (TEAEs) was similar between patients with earlier and longer exposure to opicapone (OPC–OPC; 78.8%) and those with later initiation (delayed by 14–15 weeks) following placebo (PLC–OPC; 80.0%), with comparable severity distributions and most events classified as mild or moderate. TEAEs leading to treatment discontinuation were reported more frequently in the OPC-OPC arm than in the PLC-OPC arm (6 [5.1%] vs. 0 [0.0%]; *p* = 0.03; *post-hoc* power estimate, 55.9%). Treatment-related TEAEs leading to discontinuation were reported in four patients in the OPC-OPC arm and none in the PLC-OPC arm, although the difference was not statistically significant (*p* = 0.12). Treatment-related dyskinesia occurred at similar cumulative rates with earlier and delayed exposure (15.3% vs. 15.5%, *p* = 1.00), with no evidence of increasing incidence over longer treatment duration (Figure 3). Back pain was reported more frequently in the delayed-start group than in the early-start group across the combined study period (2.5% vs. 10.0%; *p* = 0.04; *post-hoc* power estimate, 67.6%). This difference was not observed when the double-blind and open-label phases were analyzed separately. Small, non-significant differences between groups were observed for selected TEAEs. Although infrequent overall, worsening of parkinsonism was reported more frequently in the delayed-start group than in the early-start group (4.5% vs. 1.7%), and hallucinations (including visual) were reported more frequently in patients with earlier initiation of opicapone (5.9% versus 1.8%).

**Figure 3:**
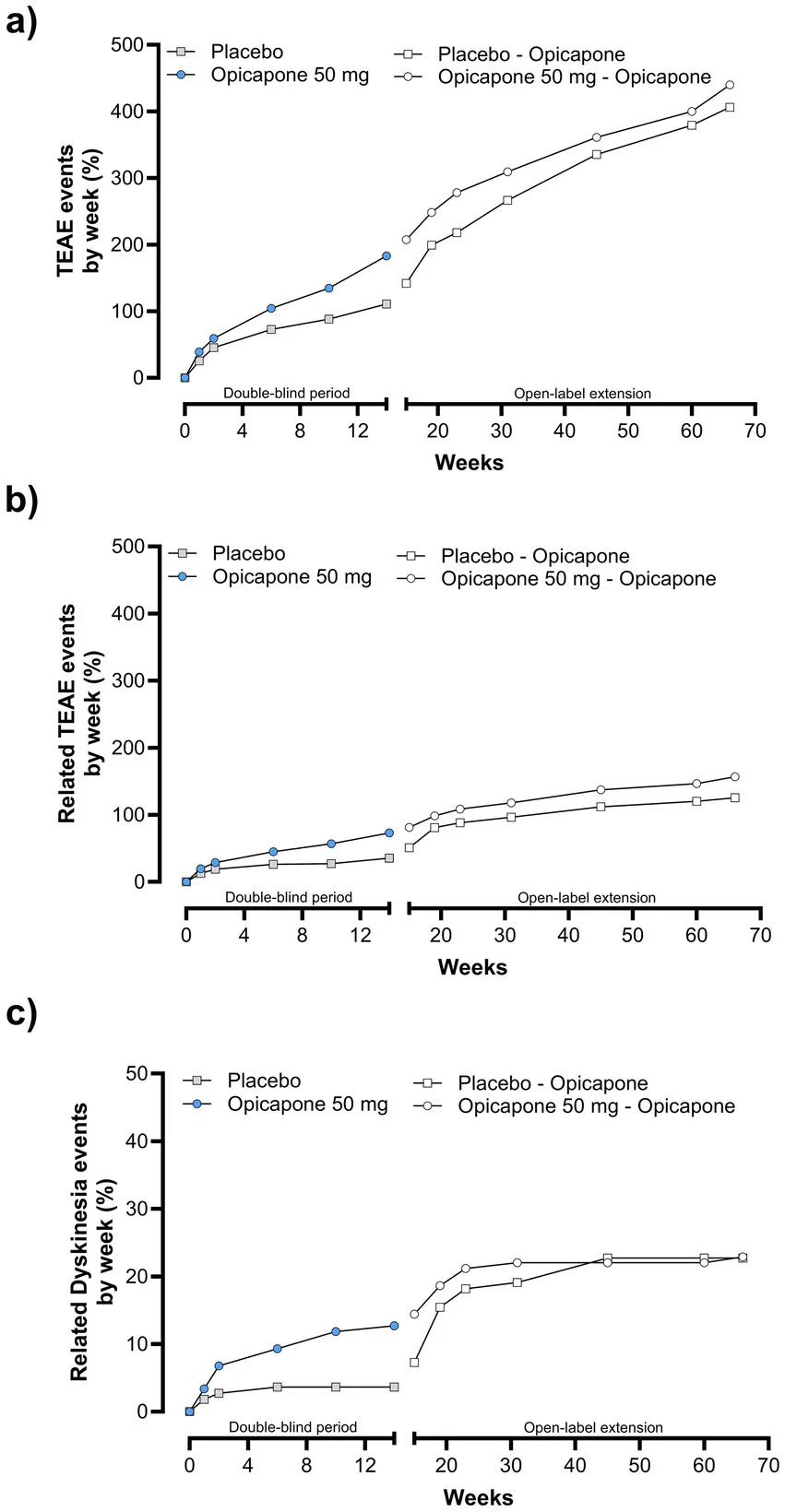
Cumulative incidence of adverse events across the double-blind and open-label phases for **(a)** Overall treatment-emergent adverse events, **(b)** Treatment-related TEAEs, and **(c)** Treatment-related dyskinesia in patients with onset of motor fluctuations ≤2 years. Graphs display the total number of events, and a single patient may have reported more than one event. Potentially related defined as definite, possible, probable or with missing information.

Other potentially related adverse events of special interest for dopaminergic therapies were infrequent and occurred at low and generally comparable rates between treatment groups. Impulse control–related events occurred in only two individuals on opicapone (pathological gambling, compulsive shopping and hypersexuality, *n* = 1 and impulsive behavior, *n* = 1). Sleep-related events were uncommon, including somnolence/sleep attacks (1.7% vs. 3.6%), nightmares (0.8% vs. 3.6%), and abnormal dreams (0.0% vs. 1.8%).

### 3.5 Safety and tolerability in a subgroup of patients with CNS comorbidities of special interest

Although BIPARK I and II excluded PwP with major neuropsychiatric and sleep comorbidities, a small subgroup of 55 patients (opicapone, *n* = 30; placebo, *n* = 25) with mild ongoing CNS comorbidities was included. The most frequent conditions were insomnia (45.5%), depression (38.2%), and anxiety (18.2%; Supplementary Table 1). In this subgroup, the overall double-blind safety and tolerability profile of opicapone was consistent with that observed in the overall early motor fluctuation population (Supplementary Table 2). During the double-blind phase, hallucination was reported in one patient (3.4%) receiving opicapone, while impulse control–related events occurred in two patients (the same cases described above). Mood-related adverse events were uncommon, with one report of depression in the placebo arm, and sleep-related events were reported at low frequencies. Overall, this exploratory subgroup analysis showed no evidence of cumulative neuropsychiatric risk or worsening of pre-existing sleep, cognitive, and/or psychiatric conditions with opicapone treatment. During the open-label phase, potentially treatment-related TEAEs were reported more frequently in the PLC-OPC arm than in the OPC-OPC arm (64.0% vs. 30.0%; *p* = 0.02; *post-hoc* power estimate, 73.1%). Dyskinesia events were also numerically more common in the PLC-OPC arm (36.0% vs. 10.0%; *p* = 0.046; *post-hoc* power estimate, 68.3%). The higher incidence of dyskinesia may reflect the greater prevalence of baseline dyskinesia among PLC-OPC patients (68% vs. 33% in the OPC-OPC arm).

## 4 Discussion

This analysis provides a detailed characterization of the safety and tolerability of opicapone in patients with recent onset of motor fluctuations (≤2 years). Across double-blind and open-label periods, the overall incidence and severity of TEAEs were low and predominantly mild to moderate, transient, and non-progressive. The early, time-limited clustering of dopaminergic adverse events, followed by stabilization, suggests a brief adaptation period rather than a persistent tolerability issue. The pattern of AEs observed in this subgroup was consistent with that of the overall parent trial population, albeit with a numerically lower incidence of TEAEs (56% vs. 63%, respectively), likely reflecting the less advanced disease stage of this ‘early fluctuating’ subgroup compared with the broader pivotal trial population (8–10). Overall, these findings are consistent with real-world observational evidence suggesting favorable tolerability with earlier use (12, 17–19).

Consistent with COMT inhibition and the associated increase in levodopa bioavailability, opicapone TEAEs were predominantly dopaminergic in nature, with dyskinesia the most frequently reported event. Dyskinesia incidence was notably lower in this early fluctuating population than in the parent studies (10% vs. 20%, respectively) (10, 20), and was similar between early and delayed initiation groups, indicating no excess risk with earlier or longer exposure. Dyskinesia typically emerged within the first 2–3 weeks of treatment and was generally mild to moderate in severity. Most cases resolved (83%), either spontaneously or following modest levodopa dose adjustment. The frequent documentation of resolution by investigators further suggests that these events did not persist, or at least they became less noticeable or better tolerated over time. In line with known risk factors, dyskinesia events were more common among patients with more advanced disease and higher levodopa exposure (1, 4). Neuropsychiatric events are also clinically relevant when evaluating dopaminergic adjunct therapies, particularly in patients with pre-existing vulnerability. In this analysis, opicapone was not associated with cumulative neuropsychiatric worsening, even in patients with mild pre-existing vulnerabilities. Although detailed descriptions of neuropsychiatric events were not systematically recorded, they were generally transient, manageable, and there was no evidence of cumulative risk over prolonged exposure. Impulse control–related events were also rare and occurred in patients with mild pre-existing psychiatric comorbidities. Consistent with this, rates of psychosis and related events in the broader opicapone clinical program (10) have been low and aligned with expectations for dopaminergic therapies. The isolated statistical significance observed for increased blood CPK during the double-blind phase (all TEAEs) was not replicated when analyses were restricted to treatment-related events or extended to longer follow-up. Similarly, the higher incidence of back pain observed in the delayed-start group over the combined exposure period was not evident within either study phase analyzed separately and lacks a clear pharmacological explanation. TEAEs leading to treatment discontinuation were more frequent with earlier opicapone initiation; however, the small number of events and the absence of a significant difference in treatment-related discontinuations specifically do not support a robust or clinically meaningful safety signal associated with treatment sequence. Collectively, these findings suggest that isolated statistically significant results should be interpreted cautiously, particularly when they are not consistently observed across related analyses, and may reflect chance findings arising from multiple comparisons.

These findings extend safety evidence from pivotal trials by providing new long-term data in patients with recent motor fluctuations, showing that opicapone maintains favorable tolerability at an earlier disease stage (with and without other PD medications). Earlier initiation was associated with a numerically lower incidence of overall adverse events, including dyskinesia, compared with broader pivotal trial populations (10), supporting the absence of an increased safety burden with longer exposure. The data do not indicate that earlier optimization with opicapone accelerates the emergence of motor complications; rather, the data support early adjunctive use without an apparent increase in dyskinesia or other dopaminergic safety concerns. Taken together with evidence for a benefit of earlier (vs later) levodopa optimization with opicapone in early fluctuators (14), the present findings support consideration of opicapone as part of early treatment optimization rather than a late add-on. From a broader clinical perspective, this aligns with growing support for earlier optimization of the levodopa-based treatment regimen (6, 7). Accumulating evidence across the disease course—from patients without motor fluctuations (21, 22) to those with early and more advanced fluctuations—suggests a consistent tolerability profile, with fewer adverse events when opicapone is initiated earlier. Earlier stabilization of levodopa exposure may also delay the need for subsequent treatment escalation and increasing therapeutic complexity, with potential implications for adherence and persistence.

### 4.1 Limitations

This analysis has several limitations. As a *post-hoc* pooled evaluation of the same clinical trial population, it shares inherent limitations of the original controlled studies, which were designed to enroll relatively homogeneous patients and may therefore limit generalizability, despite inclusion of individuals with mild comorbidities. As a subgroup analysis, all findings should be interpreted as exploratory. A key limitation is that the onset of motor fluctuations was determined by investigators based on clinical history, which may underestimate the true onset and duration of fluctuation symptoms. Potential confounding from concomitant dopaminergic therapies cannot be fully excluded, and treatment adjustments during the open-label phase may have influenced observed outcomes. The open-label extension is also subject to selection and attrition biases, as well as the absence of a placebo control, which may affect interpretation of long-term safety and tolerability. As is typical for pivotal registration studies, AE collection focused on event incidence, severity, relationship to treatment, and outcome rather than detailed clinical phenomenology; consequently, such data were not available for analysis. Any between-group differences observed in these exploratory *post-hoc* analyses should be interpreted cautiously, particularly given the small number of events and modest statistical power of some comparisons; therefore, statistically significant findings should be regarded as hypothesis-generating rather than confirmatory. Despite these limitations, the use of individual patient-level data, consistency of study design across trials, and concordance of findings between the double-blind and open-label phases support the overall robustness and internal consistency of the observed favorable safety and tolerability profile in patients with early motor fluctuations.

## Data Availability

The original contributions presented in the study are included in the article/Supplementary material, further inquiries can be directed to the corresponding author.
