# Supplementary material for: Long-term safety profile of opicapone in patients with Parkinson’s disease and early fluctuations
Source: Front Neurol. 2026 Oct 13;17:1920359. doi: 10.3389/fneur.2026.1920359 (PMC13711507; doi:10.3389/fneur.2026.1920359)
Supplement: Supplementary file 1 [file Data_Sheet_1.PDF]

Supplementary Material

Figure e1. Patient disposition through the phase 3 double-blind and open-label trials

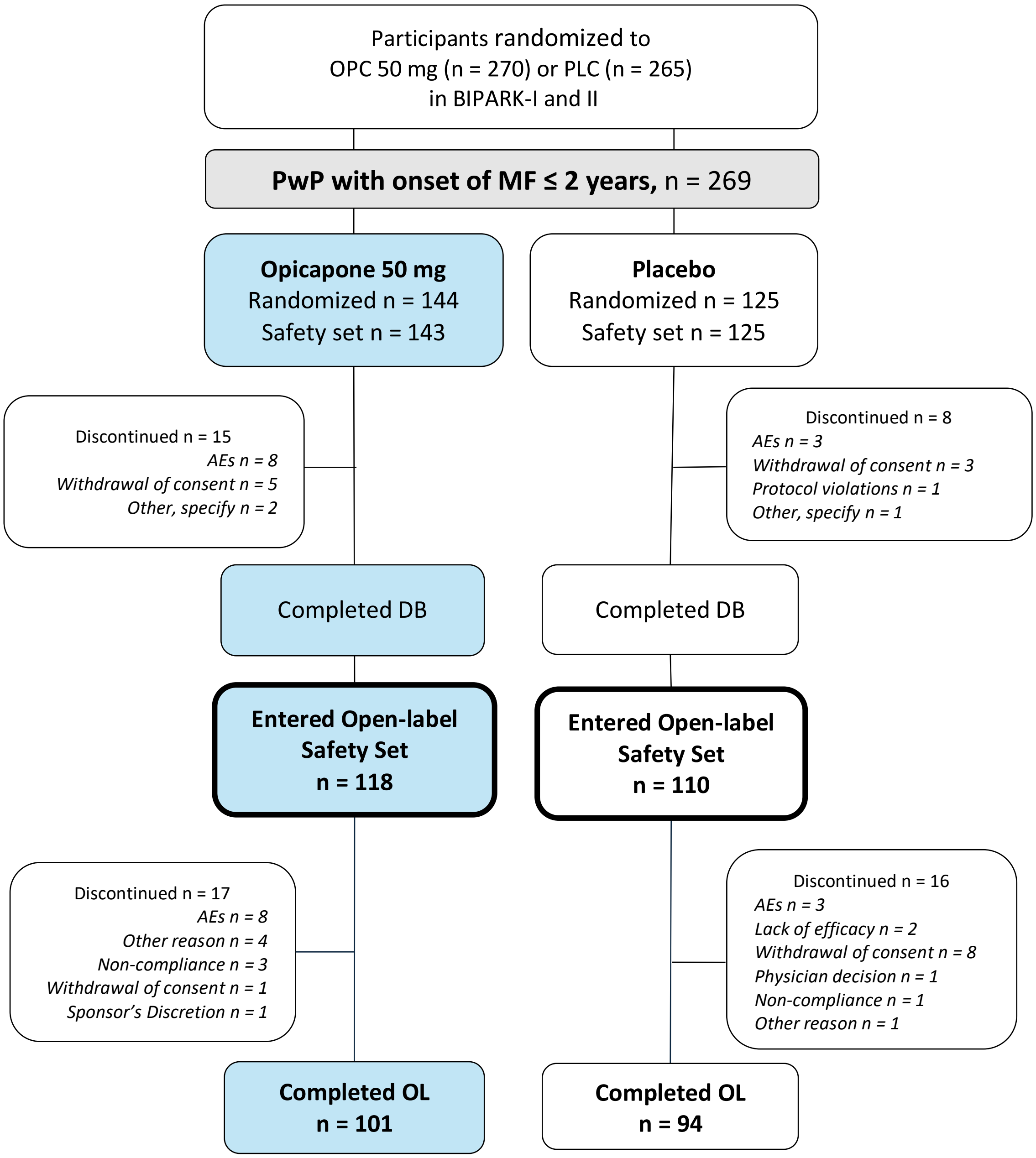

Table e1. Baseline conditions by preferred term in patients with mild* psychiatric and sleep-related comorbidities at baseline

	**Opicapone n=30**	**Placebo n=25**
Male, n (%)	18 (60.0%)	12 (48.0%)
Age, years	63.4 ± 9.2	63.8 ± 9.3
Time since PD diagnosis, years	5.6 ± 2.4	6.3 ± 2.2
Modified Hoehn & Yahr at ON, stage	2.1 ± 0.5	2.2 ± 0.6
Time since motor fluctuation onset, years	1.2 ± 0.5	1.2 ± 0.5
Presence of dyskinesia, yes, n (%)	10 (33.3%)	17 (68.0%)
**Comorbid condition***		
Insomnia	15 (50.0%)	10 (40.0%)
Depressive disorder, including depressive disorder, depressed mood, major depression	10 (33.3%)	11 (44.0%)
Anxiety disorder	6 (20.0%)	4 (16.0%)
Sleep disorder	2 (6.7%)	3 (12.0%)
Rapid eye movements sleep abnormal	3 (10.0%)	2 (8.0%)
Cognitive disorder, including Cognitive impairment, loss of memory, memory decreased, and disturbance in consciousness	2 (6.7%)	2 (8.0%)
Somnolence	1 (3.3%)	1 (4.0%)
Abnormal dreams	1 (3.3%)	1 (4.0%)
Bruxism	1 (3.3%)	0 (0.0%)
Post-traumatic stress disorder	1 (3.3%)	0 (0.0%)
Confusional arousal	0 (0.0%)	1 (4.0%)
**PD medications**		
Levodopa duration, years	4.5 ± 2.7	5.6 ± 2.5
Levodopa dose at baseline, mg/day	681 ± 353	685 ± 329
LEDD[$] at double-blind baseline, mg/day	781 ± 332	773 ± 312
LEDD[$] at open-label baseline, mg/day	1115 ± 495[£]	768 ± 290
Dopamine agonists, n (%)	21 (70.0%)	16 (64.0%)
MAO-B inhibitor, n (%)	9 (30.0%)	7 (28.0%)
Amantadine, n (%)	6 (20.0%)	4 (16.0%)
Anticholinergics, n (%)	1 (3.3%)	1 (4.0%)

**Medical Dictionary for Regulatory Activities (MedDRA classification). Patients with clinically significant psychiatric conditions (including major depression, dementia, impulse control disorders, or suicidal ideation), were excluded from pivotal trials. [$]LEDD calculated considering MAO-B inhibitors and dopamine agonists. [£]Considering OPC for LEDD calculation.*

Table e2. Safety and tolerability of opicapone in patients with mild psychiatric and sleep-related comorbidities at baseline

	Double-blind phase			Open-label phase		
	Opicapone 50 mg n=30	Placebo n=25	p-value	OPC-OPC n=30	PLC-OPC n=25	p-value
**All TEAEs, n (%)**	17 (56.7%)	18 (72.0%)	0.37[±]	21 (70.0%)	21 (84.0%)	0.37[±]
Mild	15 (50.0%)	17 (68.0%)	0.28[±]	18 (60.0%)	18 (72.0%)	0.52[±]
Moderate	11 (36.7%)	4 (16.0%)	0.16[±]	13 (43.3%)	17 (68.0%)	0.12[±]
Severe	1 (3.3%)	2 (8.0%)	0.59[$]	3 (10.0%)	4 (16.0%)	0.69[$]
Serious	0 (0.0%)	1 (4.0%)	0.45[$]	1 (3.3%)	2 (8.0%)	0.59[$]
Leading to discontinuation	0 (0.0%)	0 (0.0%)	-	3 (10.0%)	0 (0.0%)	0.24[$]
**Potentially related[$] TEAEs, n (%)**	12 (40.0%)	11 (44.0%)	0.98[±]	9 (30.0%)	16 (64.0%)	0.02 [±] (73.1%)Δ
Serious	0 (0)	1 (4.0%)*	0.45[$]	1 (3.1)[#]	0 (0)	1.00[$]
Leading to discontinuation	0 (0)	0 (0)	-	2 (6.7%)+	0 (0)	0.49[$]
**Potentially related[$] TEAEs**						
Dyskinesia	4 (13.3%)	4 (13.3%)	1.00[$]	3 (10.0%)	9 (36.0%)	0.05[±] (68.3%)Δ
Somnolence	2 (6.7%)	2 (8.0%)	1.00[$]	0 (0.0%)	2 (8.0%)	0.20[$]
Dry mouth	1 (3.3%)	2 (8.0%)	0.59[$]	0 (0.0%)	2 (8.0%)	0.20[$]
Nausea	1 (3.3%)	2 (8.0%)	0.59[$]	0 (0.0%)	2 (8.0%)	0.20[$]
Blood CPK increased	0 (0.0%)	0 (0.0%)	-	2 (6.7%)	3 (12.0%)	0.65[$]
Hypertension	1 (3.3%)	0 (0.0%)	1.00[$]	2 (6.7%)	1 (4.0%)	1.00[$]
Parkinson's disease	1 (3.3%)	1 (4.0%)	1.00[$]	1 (3.3%)	2 (8.0%)	0.59[$]
Insomnia	1 (3.3%)	0 (0.0%)	1.00[$]	0 (0.0%)	3 (12.0%)	0.09[$]
Muscle rigidity	0 (0.0%)	0 (0.0%)	-	0 (0.0%)	2 (8.0%)	0.20[$]

**Radius fracture; #Head injury; +head injury, n=1; Parkinson's Disease, n=1. Statistical comparisons were exploratory and not adjusted for multiplicity; given the number of tests performed and the limited sample size, P-values should not be interpreted as confirmatory evidence of a treatment difference [P-value were calculated using ±Chi-square test with Yates' continuity correction or $Fisher's exact test. ΔFor statistically significant findings (p < 0.05), post hoc power analysis was performed to assess the probability of detecting the observed effect].*
