# Supplementary material for: Long-term safety profile of opicapone in patients with Parkinson’s disease and early fluctuations
Source: Front Neurol. 2026 Oct 13;17:1920359. doi: 10.3389/fneur.2026.1920359 (PMC13711507; doi:10.3389/fneur.2026.1920359)
Supplement: Supplementary file 2 [file Data_Sheet_2.pdf]

List of IECs or IRBs

Including the Name of the Committee Chair

Country / Center No.	Name and address of IEC/IRB	Name of chairperson
**AUSTRIA**		
1002	Ethikkommission der Stadt Wien<br>Thomas-Klestil-Platz 8<br>A-1030 Wien	Dr. Karin Spacek
1004	Ethikkommission des Landes Oberösterreich<br>c/o Landesnervenklinik Wagner-Jauregg<br>Wagner-Jauregg Weg 15<br>A-4020 Linz	Univ.-Prof. Dr. Johannes Fischer
**BULGARIA**		
ALL SITES	Ethics Committee for Multicentre Clinical Trials<br>Administrative Services Unit at Ministry of Health<br>5 “Sveta Nedelya” Square<br>1000 Sofia	Anastas Stoikov, MD
2301	UMHAT “Alexandrovska”<br>1 St. Geogri Sofiiski Str.<br>1403 Sofia	Prof. Mila Vlaskovska
2302	CCB MI Ministry of Interior<br>79 Skobelev Blvd.<br>1606 Sofia	Assoc. prof. Dimitar Popov, MD
2304	First MHAT-Sofia<br>37 Patriarh Evtimii Bvld.<br>1000 Sofia	Assoc. prof. Zlatka Yankova
2305	MBAL "Tokuda Hospital Sofia"<br>51B Nikola Vaptzarov Bvld.<br>1407 Sofia	Assoc. prof. Ivan Staikov, MD
2307	MHAT “Prof. Stoian Kirkovich”<br>Stoletov Str.<br>6000 Stara Zagora	Rumyana Radieva, MD

Country / Center No.	Name and address of IEC/IRB	Name of chairperson
2308	Fourth MHAT – Sofia<br>38“Makedonia” Blvd.<br>1606 Sofia	Lyudmila Lyubenova, MD
2309	UMHAT "Tsaritsa Yoanna"- Sofia<br>8 "Byalo more" St.<br>1527 Sofia	Assoc. prof. Borislav Vladimirov, MD
2310	Diagnostic & Consultative Center “Sveta Anna”<br>1 Dimitar Mollov St.<br>1709 Sofia	Milkana Simeonova, MD
**CROATIA**		
ALL SITES	Agencija za lijekove i medicinske proizvode RH<br>Središnje Etičko Povjerenstvo<br>Ksaverska cesta 4<br>10 000 Zagreb	
3001	Hospital Drug Committee; Clinical Hospital Centre Split; Spinčićeva 1,<br>21 000 Split,	Contact person:<br>Marija Bekavac, dipl.iur.
3002	Hospital Drug Committee, Clinical Hospital Centre Zagreb, Kišpatićeva 12,<br>10 000 Zagreb	Contact person:<br>Nika Kalogjera, MD
3004	Hospital Drug Committee, Clinical Hospital Osijek,<br>J. Huttlera 4,<br>31 000 Osijek	Contact person:<br>Suzana Mimica Matanović, MD
3003	Ethics Committee Sveti Duh, Clinical Hospital Sveti Duh, Sveti Duh 64,<br>10 000 Zagreb<br>Drug Committee Sveti Duh, Clinical Hospital Sveti Duh, Sveti Duh 64,<br>10 000 Zagreb,	Prof. Marija Graberski Matasović, PhD<br><br>Ivana Mikačić, MD (since January 2013)

Country / Center No.	Name and address of IEC/IRB	Name of chairperson
3005	Hospital Ethics Committee, General Hospital Zadar, B. Šimurine 5,<br>23 000 Zadar<br>Hospital Drug Committee, General Hospital Zadar,<br>B. Šimurine 5,<br>23 000 Zadar	Tanja Šimurina, MD<br><br>Assistant Profesor Aleksandar Kanežević, MD PhD
**CZECH REPUBLIC**		
3103	LEC of NZZ Clintrial<br>Počernická 1427/16<br>100 00 Praha 10	Jaroslav Hofman, JUDr
3104	LEC of Region Hospital Pardubice<br>Kyjevská 44<br>532 03 Pardubice	Josef Hájek, MD
3105	LEC Municipal Hospital Ostrava<br>Nemocniční 20<br>728 80 Ostrava	Jan Nieslanik, MD
3102	LEC of Orlickoústecká Hospital<br>Čs. Armády 1076<br>562 18 Ústí nad Orlicí	Miroslav Kareš, MD
3109	LEC Fakultní nemocnice Olomouc<br>I.P.Pavlova 6<br>775 20 Olomouc	doc. MUDr. Vladko Horčička, CSc.
3108	LEC Všeobecné fakultní nemocnice v Praze<br>Na Bojišti 1<br>128 08 Praha 2	MUDr. Josef Šedivý, CSc.
3106	LEC NZZ Clintrial, s.r.o<br>Počernická 1427/16<br>100 00 Praha 10	Jaroslav Hofman, JUDr.
3107	LEC Fakultní nemocnice u sv. Anny v Brně<br>Pekařská 53<br>656 91 Brno	MUDr. Vladimír Soška, CSc.

**Country / Center No.**	**Name and address of IEC/IRB**	**Name of chairperson**
MEC<br>ALL SITES	LEC of University Hospital Brno<br>Jihlavská 20<br>625 00 Brno	PharmDr.Šárka Kozáková
**FRANCE**		
ALL SITES	Comités de Protection des Personnes Sud Ouest et Outre Mer I et Sud Ouest et Outre Mer II<br>Agence Régionale de Santé MidiPyrénées, Bureau 1028<br>10 chemin du raisin<br>31050 Toulouse Cedex 9	Mr. M. Riviere
**GERMANY**		
LEADING EC<br>ALL SITES	Kommission für Ethik in der ärztlichen Forschung des Fachbereichs Humanmedizin der Philipps-Universität Marburg<br>Baldingerstraße/Postfach 2360,<br>35033 Marburg	Prof. Dr. med. Gerd Richter
1202	Ethik-Kommission der Medizinischen Hochschule Hannover<br>Carl-Neuberg-Straße 1,<br>30625 Hannover	Prof. Dr. H. D. Tröger
1204	Ethik-Kommission der Fakultät für Medizin der Technischen Universität München<br>Ismaninger Str. 22,<br>81675 München	Contact person: Prof. Dr. G. Schmidt
1205	Ethik-Kommission der Medizinischen Fakultät der Eberhard-Karls-Universität und am Universitätsklinikum Tübingen<br>Gartenstaße 47 , 72074 Tübingen	Prof. Dr. med. Dr. Dieter Luft
1210	Ethikkommission der Landesärztekammer Brandenburg<br>Hauptgeschäftsstelle<br>Dreifertstraße 12<br>03044 Cottbus	

Country / Center No.	Name and address of IEC/IRB	Name of chairperson
1211	Ethik-Kommission der Landesärztekammer in Hessen<br>Im Vogelsgesang 3,<br>60488 Frankfurt	Prof. Dr. med. Sebastian Harder<br>Prof. Dr. med. Hannsjürgen Bratzke
1207	Ethikkommission der Medizinischen Fakultät der Ruhr-Universität Bochum<br>BG-Universitätsklinikum Bergmannsheil GmbH, Bürkle-de-la-Camp-Platz 1;<br>44789 Bochum	Prof. Dr. med. Michael Zenz
1206	Ethik-Kommission der Fakultät für Medizin der Technischen Universität München<br>Ismaninger Str. 22,<br>81675 München	Prof. Dr. A. Schömig
1213	Geschäftsstelle der Ethik-Kommission des Landes Berlin<br>Landesamt für Gesundheit und Soziales (LAGeSo)<br>Fehrbelliner Platz 1,<br>10707 Berlin	
1216	Ethikkommission der Otto-von-Guericke-Universität an der Medizinischen Fakultät um am Universitätsklinikum Magdeburg A.ö.R.<br>Leipziger Str. 44,<br>39120 Magdeburg	
**HUNGARY**		
ALL SITES	Central Ethics Committee<br>Medical Research Council<br>Ethics Committee for Clinical Pharmacology<br>(Egészségügyi Tudományos Tanács<br>Klinikai Farmakológiai Etikai Bizottsága)<br>Arany J. u. 6-8<br>1051 Budapest	

Country / Center No.	Name and address of IEC/IRB	Name of chairperson
**ITALY**		
CEC, 4101	Comitato Etico<br>Ospedale San Raffaele<br>Via Olgettina, 60<br>20132 Milano	Ref. Dr. Elisabetta Riva
4102	Nucleo per la Ricerca Clinica dell'ULSS 12<br>Via Don F. Tosatto, 147<br>30124 Mestre (VE)	Dr. Nicolò Anesa
4103	Comitato Etico<br>dell'Azienda Policlinico umberto I di Roma<br>Viale del Policlinico, 155<br>00161 Roma	
4107	Comitato Etico dell' Ospedale San Martino di Genova<br>Largo Rosanna benzi, 10<br>16132 Genova (Italy)	Ref. Paola Freccia
4109	Comitato Etico dell'IRCCS S. Raffaele Pisana di Roma<br>Via della Pisana 235<br>00163 ROMA	Secretariat:<br>Mrs Fabiola D'Angeli
**LATVIA**		
ALL SITES	Ethics Committee for Clinical Research at Development Society of Pauls Stradins Clinical University Hospital<br>Pilsonu 13,<br>Riga, LV- 1002	
**LITHUANIA**		
ALL SITES	Central EC<br>Lithuanian Bioethics Committee<br>Didzioji St. 22<br>LT-01128 Vilnius	

Country / Center No.	Name and address of IEC/IRB	Name of chairperson
5102, 5104	Vilnius Regional Bioethics Committee<br>M. K. Ciurlionio St. 21/27 (room No. 231)<br>LT-03101 Vilnius	
5101, 5107	Valid from 26.11.2013: Kaunas Regional Bioethics Committee<br>Hospital of Lithuanian University of Health Sciences<br>Mickevičiaus 9, Room# 205 (Department of Physiology)<br>LT-50009 Kaunas	
**POLAND**		
ALL SITES	Bioethics Committee at Local Medical Chamber<br>ul. Krupnicza 11A,<br>31-123 Krakow	dr. Mariusz Janikowski
**PORTUGAL**		
ALL SITES	CEIC: Comissão de Ética para a Investigação Clínica<br>Parque de Saúde de Lisboa, Avenida do Brasil nº 53 - Pav 17 A<br>1749-004 Lisboa	Prof. Dr. Alexandre Quintanilha
**ROMANIA**		
ALL SITES	Valid from Feb 2014: Comisia Nationala de Bioetica a Medicamentului si a Dispozitivelor Medicale<br>Sos. Stefan cel Mare, nr. 19-21, sector 2,<br>020125, (pavilion K, Spital Colentina), Bucuresti	President: Ac.Prof.Dr.Dinu Antonescu
**RUSSIA**		
ALL SITES	Central EC: Council on Ethics<br>3, Rakhmanovsky per.,<br>127994, Moscow	

Country / Center No.	Name and address of IEC/IRB	Name of chairperson
5305	Committee on Ethics at the Federal State Budget Institution of Sciences "N.P. Bechtereva Institute of the Human Brain of the Russian Academy of Sciences"<br>9, Acad. Pavlova, St. Petersburg, 197376	
5306	Independent Committee on Ethics under the State Budget Institution of Public Health of Moscow region “Moscow Regional Research Clinical Institute named after M.F. Vladimirsky”<br>61/2, Schepkina str., Moscow, 129110	
5307	Ethics Committee at the State Budget Institution of Public Health of Nizhniy Novgorod region "Nizhniy Novgorod Regional Hospital named after N.A. Semashko"<br>190, Rodionova str., Nizhniy Novgorod, 603126	
5308	Regional Ethics Committee at the State Medical Institution "Kursk State Medical University"<br>3, K. Marks str., 305041, Kursk	
5309	Local Ethic Committee at the Regional State Budget Institution of Public Health "Belgorod Regional Clinical Hospital of Sant Joosaf"<br>8/9, Nekrasova str., Belgorod, 308007	
5310	Ethics Committee at the Federal State Budget Institution “State Scientific Center of the Russian Federation - Federal Medical Biophysical Center n.a. A.I. Burnazian”<br>46, Zhivopisnaya, Moscow, 123182	

Country / Center No.	Name and address of IEC/IRB	Name of chairperson
**SERBIA**		
2001	Clinical Centre of Serbia Ethics Committee<br>2. Pasterova St.<br>11000 Belgrade	Contact person: Ms Branka Krunic
2002	Military Medical Academy Ethics Committee<br>17. Crnotravska St.<br>11000 Belgrade	Contact person: MSc New: Ms Nada Cikaric
2003	Clinical Centre Nis Ethics Committee<br>48. Dr Zorana Djindjica Blvd.<br>18000 Nis	Contact person: Mr Aleksandar Lazarevic
2004	Clinical Centre of Vojvodina Ethics Committee<br>1. Hajduk Veljkova St.<br>21000 Novi Sad	Contact person: Ms Dusica Kremenovic
2005	Clinical Centre Kragujevac Ethics Committee<br>30. Zmaj Jovina St.<br>34000 Kragujevac	Contact person: Ms Vera Nastic
**SLOVAKIA**		
3205	LEC Trnavského samosprávneho kraja<br>P.O.BOX 128, Starohájska 10<br>917 01 Trnava	since May 2014: Zuzana Fuňáková
CEC, 3201	LEK Trenčianskeho samosprávneho kraja<br>K dolnej stanici 7282/20A<br>911 01 Trenčín	Doc. MUDr. Ján Bielik, CSc
3202	Etická komisia Žilinského samosprávneho kraja<br>Komenského 48<br>011 09 Žilina	MUDr. Martin Kapasný
3203, 3204	Nezávislá etická komisia Banskobystrického samosprávneho kraja<br>Námestie SNP 23<br>974 01 Banská Bystrica	Mgr. Katarína Čupková

Country / Center No.	Name and address of IEC/IRB	Name of chairperson
**SPAIN**		
4301	Secretaria Comité Ético de Ensayos Clínicos IMIM<br>Parc de Recerca Biomédica de Barcelona<br>C/ Doctor Aiguader, 80<br>08003 Barcelona	Contact person: Mª Teresa Navarra Alcrudo
4302	Comité de Ensayos Clíncos Hospital de la Santa Creu i Sant Pau<br>Servicio de Farmacología Clínica<br>Pavellón nº 19<br>Avinguda Sant Antoni Maria Claret, 165<br>08025 Barcelona	Contact persons: Marcela Domínguez/Albert Querol
4303	CEIC Institut Universitari Dexeus<br>ecretaría del Comité Ético de Ensayos Clínicos<br>Comité Ético del Institut Universitari Dexeus<br>Sabino Arana 5-19<br>08028 Barcelona	Contact person: Carme Garcia
4304	CEIC HUVH-Agencia de Ensayos Clínicos<br>Edifici Institut de Recerca (2ª Planta)<br>Hospital Universitari Vall d'Hebron<br>Passeig Vall d'Hebron 119-129<br>08035 Barcelona	Contact person: Teresa Navarra Mireia Navarro
4305	Comité Ético de Ensayos Clínicos Hospital Clínic i Provincial de Barcelona<br>Agencia de Ensayos Clínicos. Servicio Farmacia<br>Villarroel, 170 - Sótano, Escalera 6b<br>08036 Barcelona	Contact person: Alicia Bernal

Country / Center No.	Name and address of IEC/IRB	Name of chairperson
4306	Comité de Ensayos Clíncos Hospital Universitario Virgen Macarena<br>Avda. Dr. Fedriani, 3. 41009 – Sevilla<br><br>Comité de Ensayos Clínicos Autonómico de E.C de Andalucía<br>CEIC Autonómico de E.C de Andalucía<br>Avda. Innovación, s/n Edif. Arena 1.<br>41020 – Sevilla	Contact person: Carlos García Pérez<br><br>Contact person: Mª Angeles Rasero
4307	Comité Ensayos Clínicos Hospital Ruber Internacional<br>C/ La Masó, 38<br>28034 Madrid	Contact person: Dr.García Labajo
4309	CEIC Hospital General de Catalunya<br>Calle Pere y Pons, s/n<br>08195 Sant Cugat del Valles	Contact person: Montse Granados
4311	Comité de Ensayos Clíncos Hospital Universitario La Paz<br>Hospital General-Planta 8<br>Despachos 218-219<br>Paseo de la Castellana,261<br>28046 Madrid	Contact persons: Dra. Paz Lavilla /<br>Dra. Emma Fernández de Uzquiano
4312	Comitè Ètic d'Investigació Clínica de les Illes Balears (CEIC-IB)<br>Secretaria del Comitè Ètic d'Investigació Clínica de les Illes Balears (CEIC-IB) Conselleria de Salut i Consum-Direcció General d'Avaluació i Acreditació<br>C/ Cecili Metel, 18<br>07003 Palma - Illes Balears	Contact person: Lourdes de la Vega
**UKRAINE**		
CEC	Central Commission on Ethics Questions of Ministry of Health of Ukraine<br>Narodnogo opolchenija str. 5<br>03680 (03151) Kiev	Prof. Vasyl Mykhaylovych Kornatsky

Country / Center No.	Name and address of IEC/IRB	Name of chairperson
5201	LEC of State Institution "Institute of Gerontology NAMS Ukraine"<br>Vyshgorodskaya Str. 67<br>04114 Kiev	Prof. Valerii Shatilo
5202	LEC of Ukrainian State Scientific Research Institute of Medical and Social Problems of Disability<br>Radyasnki bystr. 1a<br>49027 Dnipropetrovsk	Dr. Yuriy Korobkin, MD, PhD
5203	LEC of SI “Institute of Neurology, Psychiatry and Narcology of NAMS of Ukraine”<br>Academician Pavlov str., 46<br>61068 Kharkiv	doc.med.scienses Lyudmyla Tantsura
5204	LEC of Communal setting of medical care Kharkiv’s regional psychiatric clinical hospital No3<br>Academician Pavlov str., 46<br>61068 Kharkiv	Dr. Konstantin Kostyrev
5205	LEC of Ternopil Regional Communal Clinical Psycho-Neurological Hospital<br>Trolleybusna Str., 14<br>46027 Ternopil	Oleksandr Bondarenko
5206	LEC of Poltavska Regional Clinical Hospital Named After M.V.Sklifosovskii<br>Schevchenko Str. 23<br>36024 Poltava	Dr. Iryna Kolomoets
5207	LEC of Communal setting City clinical hospital #2, Zaporizhzhya<br>Brullova str. 6<br>69068 Zaporizhzhya	Dr. A. Mitsik, MD, PhD
5208	LEC of Donetsk Regional Clinical Territorial Medical Unit<br>Illicha Avenue, 14<br>83003 Donetsk	Dr. Andrei Sagalevych

Country / Center No.	Name and address of IEC/IRB	Name of chairperson
**MONTENEGRO**		
2201	Scientific Review Board "CEZANA" (orig. Centar Za Nauku)/Ethics Committee<br>Clinical Centre of Montenegro<br>BB Ljubljanska St.<br>20000 Podgorica	Contact person: Ms Irena Nisavic
**BOSNIA AND HERZEGOVINA**		
2101	Clinical Centre Banja Luka<br>Ethics Committee<br>BB. 12 Beba St.<br>78000 Banja Luka	Contact person: Mr Darko Vujanovic
2102	Clinical Centre Sarajevo<br>Ethics Committee<br>25. Bolnicka St.<br>71000 Sarajevo	Contact person: Assistant professor Ms Jasmina Krehic MD PhD

Independent Ethics Committees/Institutional Review Boards consulted

Argentina

Central Ethics Committee

Centre No	Name and address of IEC/IRB	Chairman of IEC/IRB
1002	Comite de Etica en Investigacion Isntituto de Investigaciones Clinicas<br>Av Colon 3364<br>Mar del Plata	Jesus Vazquez
1005	Comite de Evaluacion Etica y Cientifica del Departamento de Docencia<br>Av Belgrano 2975<br>Cidad Autonoma de Buenos Aires<br>C1209AAB	Luis Maria Zieher
1006	Comité Independiente de Ética para Ensayos en Farmacología Clínica<br>J. E. Uriburu 774 Piso 1<br>CABA<br>Ciudad Autonoma Buenos Aires<br>C1027AAP	Luis Maria Zieher
1008	Comite de Etica en Investigacion del Instituto Medico Especializado<br>Hidalgo 568<br>Ciudad Autonoma de Buenos Aires<br>C1405BCH	Maria Victoria Bertolino
1009	Comité Independiente de Ética para Ensayos en Farmacología Clínica<br>J. E. Uriburu 774, 1st Floor<br>Ciudad Autónoma de Bs. As.<br>C1027AAP	Luis Maria Zieher

Argentina

Local Ethics Committees

Centre No	Name and address of IEC/IRB	Chairman of IEC/IRB
1004	Comite Independiente de Etica e Investigacion Biomedica<br>Uruguay 840<br>Ciudad Autonoma de Buenos Aire<br>C1015ABR	Mariana Bendersky
1005	Comite de Evaluacion Etica y Cientifica del Departamento de Docencia<br>Av Belgrano 2975<br>Cidad Autonoma de Buenos Aires<br>C1209AAB	Alicia Miranda
1006	Comite de Docencia e Investigacion del Instituto<br>Av Entre Rios 2144<br>Ciudad autónoma de Buenos Aires, 1133	Aldo Alberto Calzinari
1007	Comite de Bioetica de la Fundacion Alfredo Thomson<br>Av Diaz Velez 3992<br>Ciudad Autonoma de Buenos Aire<br>C1200AAT	Fernando Adolfo Alvarez
1008	Comite de Revision Institucional<br>Hidalgo 568<br>Ciudad Autonoma de Buenos Aires<br>C1405BCH	Amado Jose Bechara

Australia

Central Ethics Committee

Centre No	Name and address of IEC/IRB	Chairman of IEC/IRB
1102; 1103;<br>1105; 1108	Austin Health Human Research Ethics Committee<br>Research Ethics Unit, Room 8322<br>Level 8, Harold Stokes Building<br>145 Studley Road<br>Victoria, 3084	David Taylor
1106; 1109	St Vincents Hospital Research Governance Office<br>St Vincents Hospital<br>390 Victoria Street<br>Darlinghurst<br>New South Wales, 2010	Prof Jo-Anne Brien

Local Ethics Committees

Centre No	Name and address of IEC/IRB	Chairman of IEC/IRB
1101	Bellberry Human Research Ethics Committee<br>34 Brougham Street<br>East Gosford<br>New South Wales, 2250	Michael James
1102	Melbourne Health Research Governance<br>The Royal Melbourne Hospital - city campus<br>300 Grattan Street<br>Parkville, Victoria, 3050	Prof Peter Colman
1103	Austin Health Research Governance<br>145 Studley Road<br>Heidelberg<br>Victoria, 3084	David Taylor
1104	Royal Adelaide Hospital Research Ethics<br>Committee<br>Research Ethics Committee<br>Level 3, Hanson Institute, North Terrace<br>Adelaide, South Australia, 5000	A Thorton

Australia

Local Ethics Committee - Continued

Centre No	Name and address of IEC/IRB	Chairman of IEC/IRB
1105	St Vincent's Melbourne Research Governance Unit<br>Research Governance Unit<br>PO Box 2900<br>FITZROY, Victoria, 3065	Ms Kelly Hoffman
1106	Sydney West Area Health Research Governance<br>Research Office, Level 6, de Lacy Building<br>390 Victoria Street<br>Darlinghurst<br>New South Wales, 2010	Julie Charlton
1108	Southern Health Research Governance<br>Southern Health<br>246 Clayton Rd<br>CLAYTON<br>Victoria, 3168	Dr Sally Middleton
1109	St Vincents Hospital Human Research Ethics Committee<br>Level 6 de Lacey Building<br>390 Victoria Street<br>Darlinghurst<br>New South Wales, 2010	Ms Julie Charlton

Belgium

Central Ethics Committee

Centre No	Name and address of IEC/IRB	Chairman of IEC/IRB
1202- 1206	Ethisch Comité UZA (CEC)<br>Wilrijkstraat 10<br>Edegem, 2650	Prof. Dr Patrick Cras

Local Ethics Committees

Centre No	Name and address of IEC/IRB	Chairman of IEC/IRB
1202	Med Ethische Commissie H-Hartziekenhuis<br>Roeselare-Menen (LEC)<br>Wilgenstraat 2<br>Roeselare, 8800	Dr L. Marcelis
1203	Ethische Toetsingscommissie Jessa Ziekenhuis<br>Stadsomvaart 11<br>Hasselt, 3500	Dr Koen Magerman
1204	Commission d'Ethique Biomédicale Hospitalo-<br>Facultaire de l'UCL<br>Avenue Hippocrate 55.14, Tour Harvey - niveau 0<br>Bruxelles, 1200	Prof. Dr Maloteaux
1205	Ethisch Comité UZA (LEC)<br>Wilrijkstraat 10<br>Edegem, 2650	Prof. Dr Patrick Cras
1206	Lokale Commissie voor Ethiek Sint-<br>Andriesziekenhuis (LEC)<br>Krommewalstraat 9-11<br>Tielt, 8700	Dr Jozef Verhamme

Chile

Central Ethics Committee

Centre No	Name and address of IEC/IRB	Chairman of IEC/IRB
1301	Comite de Etica de la Investigacion SSM Norte<br>Calle San José 1053<br>Independencia, Santiago	Dr Carlos Navarro
1302	Comite Etico-Cientifico SS Viña del Mar-Quillota<br>Calle Limache 1307<br>Esquina Peñablanca 2°, Viña del Mar	Paola Fossa Corvalan
1303	Comite Etico Cientifico SSM Sur-Oriente<br>Av. Concha y Toro 3459<br>Puente Alto, Santiago	Dr Patricio Michaud
1304	Comite Etico-Cientifico SSM Central<br>Santa Rosa 1234, Santiago	Dr Emiliano Soto Romo
1305	Comite Etico-Científico SSM Oriente<br>Av. Salvador 364<br>Providencia, Santiago	Dr Andrés Stuardo
1306	Comite Etico- Cientifico SSM Sur<br>Santa Rosa 3453, Parad.5<br>San Miguel, Santiago	Dr Verónica Rivera
1307	Comite Etico de Investigacion SS Valdivia<br>Maipu 550, Piso 3, Of.307<br>Valdivia	Dr Ginette Grandjean Obando
1308	Comite Etico-Científico SSM Oriente<br>Av. Salvador 364<br>Providencia, Santiago	Dr Andrés Stuardo

Local Ethics Committees

Centre No	Name and address of IEC/IRB	Chairman of IEC/IRB
1304	Comité de Ética en Inv, Esc. Med, PUC<br>Marcoleta 391, Piso1, Santiago	Dr Eduardo Guarda

Czech Republic

Central Ethics Committee

Centre No	Name and address of IEC/IRB	Chairman of IEC/IRB
1401; 1402; 1404; 1406	Eticka komise Vseobecne fakultni nemocnice v Praze<br>Na Bojisti 1<br>Prague, 128 08	Josef Sedivy, MD, PhD

Local Ethics Committees

Centre No	Name and address of IEC/IRB	Chairman of IEC/IRB
1401	Lokalni eticka komise Mestske nemocnice Ostrava<br>Nemocnicni 20<br>Ostrava, 728 80	Jan Nieslanik, MD
1402	Eticka komise Centrum neurologicke pece s.r.o.<br>Jiraskova 1389<br>Rychnov nad Kneznou, 516 01	Radomir Strupl, MD
1404	Eticka komise Vseobecne fakultni nemocnice v Praze<br>Na Bojisti 1<br>Prague, 128 08	Josef Sedivy, MD, PhD
1406	Eticka komise Vseobecne fakultni nemocnice v Praze<br>Na Bojisti 1<br>Prague, 128 08	Josef Sedivy, MD, PhD

Estonia

Central Ethics Committee

Centre No	Name and address of IEC/IRB	Chairman of IEC/IRB
1501 - 1504	Tallinn Medical Research Ethics Committee<br>Hiiu 42<br>Tallinn<br>11619	J. Põlluste

India

Local Ethics Committees

Centre No	Name and address of IEC/IRB	Chairman of IEC/IRB
1702	Vidyasagar Institute of Mental Health & Neurosciences<br>1, Institutional Area<br>Nehru Nagar<br>New Delhi, 110 065	Mr Ravindra Bana
1703	Institutional Ethics Committee<br>Sri Aurobindo Seva Kendra, 1H,<br>Gariahat Road South<br>Kolkata<br>West Bengal	Mrs Manjula Bose
1704	Ethics Committee of Manipal Hospital & Manipal Heart Foundation<br>Airport Road<br>Bangalore, 560 017	Dr Justice SR Bannurmath
1705	Mallikatta Ethical Committee<br>Mamatha Residency,<br>No. 305, 2-15-1265/29, Bejai,<br>Anegundi, 1st Cross Road<br>Mangalore<br>Karnataka, 575004	Professor N Krishnamoorthy
1706	Lalitha Super Specialities Hospital Ethics Committee<br>Lalitha Super Specialities Hospital (P) Ltd, Heart & Brain Centre, Kothapet,<br>Guntur<br>Andhra Pradesh, 522001	Dr C Nageswararao
1707	Ethics Committee<br>Sahyadri Hospitals, 30 C, Erandwane<br>Karve Road<br>Pune<br>Maharashtra, 411004	Dr BG Sahastrabudhe

India

Local Ethics Committees - Continued

Centre No	Name and address of IEC/IRB	Chairman of IEC/IRB
1708	Global Independent Ethics Committee<br>1-C, Bhuyandev Scoiety<br>Near Bhuyandev Croos road, Ghatlodia<br>Ahmedabad<br>Gujarat, 380061	Mr Shashikant Sharma
1709	Kovai Medical Center and Hospital Ethics Committee<br>Kovai Medical Center and Hospital Limited<br>Post Box No. 3209, Avanashi Road, Coimbatore<br>Tamilnadu, 641 014	Dr Muthuswamy PR

Israel

Local Ethics Committees

Centre No	Name and address of IEC/IRB	Chairman of IEC/IRB
1601	Rabin Medical Center Helsinki Committee<br>39 Jabutinsky st.<br>Petach Tikva<br>49100	Prof Meir Lahav
1602	The Chaim sheba Medical Cnter Helsinki Committee<br>Tel Hashomer<br>Ramat Gan<br>52621	Prof Dror Haretz
1605	Rambam Medical Center Helsinki Committee<br>12 Aliya st.<br>Bat Galim<br>Haifa	Prof Moshe Barnet
1606	Tel Aviv Sourasky Ethics committee<br>6 Weizman st<br>Tel Aviv, 64239	Prof Marsel Topilasky

Russia

Central Ethics Committee

Centre No	Name and address of IEC/IRB	Chairman of IEC/IRB
1801 – 1804;<br>1807	Ethical Council at the Ministry of Health and Social Development of RF<br>3, Rakhmanovskyi lane<br>Moscow, 127994	Ramenskaya G.V.

Local Ethics Committees

Centre No	Name and address of IEC/IRB	Chairman of IEC/IRB
1801	EC of Siberian State Medical University of MoH and SD of RF<br>2, Moskovsky trakt<br>Tomsk, 634050	Bukreeva E.B.
1802	Ethics and Evidence Committee of "Kemerovo Regional Clinical Hospital"<br>22, Oktyabrsky prospect<br>Kemerovo, 650066	Khimchenko L.A.
1803	Ethics Committee of "Territorial Clinical Hospital"<br>1, Lyapidevskogo str<br>Barnaul, 656024	Berestov S.A.
1804	EC of SEI HPE KrasSMU named after Prof. V.F. Voyno-Yasenetsky<br>1, Partizana Zheleznyaka str<br>Krasnoyarsk, 660022	Demko I.V.
1807	Ethcs Committee at the NSMU<br>52, Krasny prospect<br>Novosibirsk, 630091	Poteryaeva E.L.

South Africa

Central Ethics Committee

Centre No	Name and address of IEC/IRB	Chairman of IEC/IRB
1902 – 1904;<br>1906 – 1908;<br>1910 - 1911	Pharma-Ethics (Pty) Ltd<br>123 Amcor Road<br>Lyttelton Manor<br>Pretoria<br>Gauteng, 0157	Dr CSJ Duvenage

Local Ethics Committees

Centre No	Name and address of IEC/IRB	Chairman of IEC/IRB
1901	University of Stellenbosch Ethics<br>Tygerberg<br>Cape Town<br>Western Cape<br>7505	Dr JAM de Roubaix
1905	University of Pretoria Ethics<br>31 Bophela Road,HW Snyman South Building<br>Level 2-Rooms 2.33, 2.34 & 2.35<br>Pretoria<br>Gauteng<br>0001	Dr CSJ Duvenage

South Korea

Local Ethics Committees

Centre No	Name and address of IEC/IRB	Chairman of IEC/IRB
2001	Samsung Medical Center IRB<br>50, Irwon-Dong, Gangnam-Gu<br>Seoul, 135-710	Lee, Suk-Koo
2002	Asan Medical Center IRB<br>388-1 Pungnap-2 dong, Songpa-gu<br>Seoul, 138-736	Jong Woo Chung
2003	Dong-A University Medical Center IRB<br>Dongdaesindong 3ga-1, Seo-gu<br>Busan, 602-715	Lee, Soo-il
2004	Severance Hospital IRB<br>250 Seongsan-no, Seodaemun-gu,<br>Seoul, 120-752	Seung Min Kim

UK

Central Ethics Committee

Centre No	Name and address of IEC/IRB	Chairman of IEC/IRB
2101 - 2102;<br>2104 - 2105	Southampton & South West Hampshire REC<br>University of Reading<br>London Road<br>Reading<br>RG1 5AQ	Dr Iain MacIntosh
